# Pure endoscopic subfrontal keyhole approach for planum sphenoidale meningiomas and olfactory groove meningiomas

**DOI:** 10.3171/2025.10.FOCVID25145

**Published:** 2026-01-01

**Authors:** Hiroki Morisako, Michael Lumintang Loe, Rayford Hazunga, Atsufumi Nagahama, Masaki Ikegami, Takeo Goto

**Affiliations:** 1 Department of Neurosurgery, Osaka Metropolitan University Graduate School of Medicine, Osaka, Japan;; 2 Department of Neurosurgery, Faculty of Medicine, University of Palangka Raya/Siloam Hospital, Dhirga Surya, Medan, Indonesia; and; 3 Doctoral Program of Medical Science, Faculty of Medicine, University of Airlangga, Indonesia

**Keywords:** planum sphenoidale meningiomas, olfactory groove meningiomas, pure endoscopic keyhole approach, subfrontal approach

## Abstract

The standard treatment for planum sphenoidale meningiomas (PSMs) and olfactory groove meningiomas (OGMs) is resection. Traditional transcranial approaches and a variety of minimally invasive techniques, such as endoscopic endonasal and endoscope-assisted subfrontal approaches, have risen in popularity. The authors describe a surgical technique for PSMs and OGMs using a purely endoscopic subfrontal keyhole approach (PESKA). Gross-total resection was achieved in all patients using PESKA. There were no new neurological symptoms postoperatively. PESKA for PSMs and OGMs is a useful surgical technique that allows for a smaller craniotomy and faster surgery, resulting in less invasiveness for patients.

The video can be found here: https://stream.cadmore.media/r10.3171/2025.10.FOCVID25145

## Transcript

In this study, we provide our first sequential case series of removing planum sphenoidale meningiomas (PSMs) and olfactory groove meningiomas (OGMs) using a purely endoscopic subfrontal keyhole technique.

### 0:31 Introduction 1.

The most common treatment for PSMs and OGMs is removal via surgical procedures. Traditional transcranial procedures such as the pterional, orbitozygomatic, transbasal, and bifrontal approaches, among others, have seen a rise in mainstream popularity.^1^ However, devastating morbidity may ensue from frontal lobe retraction, sacrifice of the superior sagittal sinus, frontal sinus transgression, and damage to the olfactory nerve.^2^

### 1:04 Introduction 2.

In recent years, several endoscopic minimally invasive methods that have the potential to reduce the need and amount of nerve and brain retraction, decrease incision length, and accelerate patient recovery have been developed and utilized. The endoscopic endonasal approach (EEA) and the endoscope-assisted eyebrow supraorbital keyhole approach are two examples of these methods.^3–5^ We describe a surgical technique for PSMs and OGMs using a pure endoscopic subfrontal keyhole approach (PESKA).

### 1:33 Preoperative Patient Selection.

To effectively prepare for endoscopic procedures, several factors should be considered, including the vascularity and surrounding structures of the tumor, the functional status of both olfactory nerves, and the dominant hemisphere of the brain. If the tumor size is larger than 35 mm, PESKA is indicated after confirming that the tumor has no pial blood supply. When deciding whether to approach from the left or right, approach from the side with the larger tumor. Furthermore, the nondominant hemisphere side is preferred.

### 2:06 Case Illustration 1.

A 48-year-old female presented with a tumor located at the planum sphenoidale. Preoperative magnetic resonance imaging (MRI) demonstrated a heterogeneously enhanced mass in the anterior skull base area, slightly larger on the right side, with a tumor size of 18 mm. The lesion was resected through our PESKA.

### 2:27 Patient Positioning and Skin Incision.

After inserting a lumbar drain, the patient was placed in a supine position with the upper body elevated 15°. A slight rotation of the head to the contralateral side, combined with an extending motion, facilitated a gravity-assisted separation of the frontal lobe from the skull base. An incision of 5 cm was made in the right eyebrow.

### 2:49 Craniotomy and Dural Opening.

After making a 3 × 3–cm right frontal craniotomy, the most critical step of the craniotomy was drilling extradurally along the inner boundaries of the anterior skull base. This expands the surgical corridor and enhances visibility upward. Mannitol was administered during the craniotomy to reduce intracranial pressure. A linear, horizontal incision in the dura is tilted outward toward the orbit.

### 3:16 Tumor Removal.

A 4-mm rigid endoscope with a 0° angle was utilized during the procedure. The portion of the dura where the tumor was attached was coagulated first. After devascularizing the tumor, it was confirmed that the right optic nerve was compressed by the tumor from the medial side. The superior tumor capsule was separated from the frontal lobe, while the right optic nerve was carefully preserved. After the arachnoid membrane was dissected away from the normal side of the brain, the tumor was then carefully removed. A small amount of tumor had invaded into the right optic canal. Therefore, the rigid endoscope was changed to a 4 mm, 30° angle, and a diamond drill was used to remove the medial superior wall of the right optic canal. After opening the right optic canal, the small amount of remaining tumor in the optic canal was removed while ensuring the right ophthalmic artery was preserved. Finally, the tumor located from the right planum sphenoidale to tuberculum sellae was completely removed, including the tumor attachment area.

### 4:36 Closure.

To avoid the CSF from leaking out of the frontal base, fatty tissue was placed above. The dura was closed in a watertight fashion, followed by fat tissue. Any unavoidable frontal sinus opening was blocked by squeezing a piece of fat tissue through the aperture. The bone was then plated, and the wound was closed without the use of a drain.

### 5:01 Postoperative Clinical Course.

The postoperative MR images showed gross-total resection of the lesion. No new neurological symptoms were observed after surgery.

### 5:11 Case Illustration 2.

A 71-year-old female presented with a tumor located at the olfactory groove and planum sphenoidale, with a tumor size of 34 mm. The lesion was resected through our PESKA.

### 5:24 Skin Incision and Craniotomy.

The incision was made in the left eyebrow. A one-piece orbitofrontal craniotomy was done. Additional bony removal was performed by drilling extradurally along the inner boundaries of the anterior skull base to extend the surgical corridor and improve upward visibility. The endoscope was inserted after the dural incision.

### 5:45 Tumor Removal.

The tumor was internally debulked with an ultrasonic surgical aspirator after being devascularized by cauterizing as much of the base as possible. The superior tumor capsule was dissected off the frontal lobe while preserving the anterior cerebral arteries and frontopolar branches. After detachment of the posterior edge of the tumor, the left optic nerve was identified. Once the arachnoid membrane had been dissected to the normal brain side, the tumor was dissected. Detachment, debulking, and coagulation of the tumor were performed until the tumor was successfully separated from surrounding structures. After gross-total tumor removal, the bilateral optic nerves and olfactory nerves were identified.

### 6:47 Postoperative Clinical Course.

The postoperative MR images showed gross-total resection of the lesion. No new neurological symptoms were observed after surgery, including olfactory function.

### 6:58 Results.

From 2020 to 2024, PESKA was used successfully on 7 patients with PSMs and OGMs. The location of lesions was 4 at the planum sphenoidale and 3 at the olfactory groove. The patients’ average age was 61.7 years, with 6 of them being female. The average tumor size was 28.1 mm. Gross-total resection was achieved in all patients with an average operative time of 237 minutes. In all patients, the contralateral olfactory nerve was preserved during surgery. There were no postoperative complications, though 1 patient had a subdural hematoma that resolved conservatively. The incisions were done around the eyebrow, and the outcomes were satisfactory from an aesthetic standpoint. There were no postoperative radiation treatments and no recurrence.

### 7:51 Discussion 1: The Use of Endoscope for PSMs and OGMs.

In 1971, Donald Wilson was one of the first neurosurgeons to use the term "key-hole surgery" to describe minimally invasive procedures.^6^ We termed the word "keyhole" as the minimum size of the supraorbital craniotomy. In our study, we showed 7 patients who underwent surgical removal of PSMs and OGMs using the PESKA technique. This approach has the advantage of maximizing exposure while minimizing incisions to the patient’s skin, with excellent visibility of the tumor and adjacent structures, including bilateral olfactory nerves and optic canals. The endoscope’s perpendicular beam eliminates shadows surrounding objects, making it easier to see detail, and offers more room for precision instrument manipulation.^7^ We operate the endoscope using a four-hand technique. The assistant uses their left hand to handle the endoscope, while their right hand operates the suction tube or forceps. In our opinion, the use of a pure endoscope in the subfrontal keyhole approach can achieve both widespread acceptability and further development in the foreseeable future. The many advantages that come with using an endoscope form the basis of our rationale for this opinion.

### 8:33 Discussion 2: Benefit and Limitations of PSMs and OGMs.

Exceptional cosmetic results are achievable with the appropriate method and surgical detail. This method has a few drawbacks, including the potential for scalp anesthesia and the chance of cerebrospinal fluid leaking via an occult frontal sinus orifice.^8^ We did not encounter any complications from scalp anesthesia and/or CSF leak through the frontal sinus. Based on the extracranial microanatomic study of the supraorbital keyhole approach, the medial border of the incision should be made 1 cm medial to the supraorbital foramen/notch. This is because, after exiting the foramen/notch, the supraorbital nerve courses on the pericranium, and its lateral branch does not give off branches within the first 10 mm of its course superiorly. Furthermore, the angle it made with the supraorbital margin was 74° ± 3° (range 68°–80°).^9^ We used fat tissue to reconstruct the defect in the dura and frontal sinuses. Several investigations have shown that the risk of CSF leakage is greatly reduced when fatty tissue grafts are used during reconstruction.^10^

One further restriction that may be, but is not always, an absolute limitation of PESKA is when dealing with hypervascular tumors. In general, unless the eye is already blind, it’s considered too risky to be performed.

### 9:38 Conclusions.

PESKA for PSMs and OGMs was reported in detail. It permits safe dissection of the tumor with adequate visibility, satisfactory tumor removal rate, and the ability to preserve cranial nerve function with minimal complications. This approach may undergo future improvements since it permits a smaller craniotomy and a shorter surgical duration, thereby reducing the invasiveness for the patients.

## Acknowledgments

The authors indicated that Ondokusan was used for video narration.

## Disclosures

The authors report no conflict of interest concerning the materials or methods used in this study or the findings specified in this publication.

## Author Contributions

Primary surgeon: Morisako, Goto. Assistant surgeon: Morisako, Nagahama, Ikegami. Editing and drafting the video and abstract: Morisako, Loe, Hazunga. Critically revising the work: Morisako, Loe, Ikegami. Reviewed submitted version of the work: Morisako, Loe, Hazunga, Goto. Approved the final version of the work on behalf of all authors: Morisako. Supervision: Goto.

## Supplemental Information

### Patient Informed Consent

The necessary patient informed consent was obtained in this study.
